# Effects of Magnetic Nanoparticles and External Magnetostatic Field on the Bulk Heterojunction Polymer Solar Cells

**DOI:** 10.1038/srep09265

**Published:** 2015-03-18

**Authors:** Kai Wang, Chao Yi, Chang Liu, Xiaowen Hu, Steven Chuang, Xiong Gong

**Affiliations:** 1 College of Polymer Science and Polymer Engineering, The University of Akron, Akron, OH 44325, USA; 2 State Key Laboratory of Luminescent Materials and Devices, South China University of Technology, Guangzhou, 510640, P. R. China

## Abstract

The price of energy to separate tightly bound electron-hole pair (or charge-transfer state) and extract freely movable charges from low-mobility materials represents fundamental losses for many low-cost photovoltaic devices. In bulk heterojunction (BHJ) polymer solar cells (PSCs), approximately 50% of the total efficiency lost among all energy loss pathways is due to the photogenerated charge carrier recombination within PSCs and low charge carrier mobility of disordered organic materials. To address these issues, we introduce magnetic nanoparticles (MNPs) and orientate these MNPS within BHJ composite by an external magnetostatic field. Over 50% enhanced efficiency was observed from BHJ PSCs incorporated with MNPs and an external magnetostatic field alignment when compared to the control BHJ PSCs. The optimization of BHJ thin film morphology, suppression of charge carrier recombination, and enhancement in charge carrier collection result in a greatly increased short-circuit current density and fill factor, as a result, enhanced power conversion efficiency.

In recent years, bulk heterojunction (BHJ) polymer solar cells (PSCs) composed of conjugated polymers (as the electron donor, D) and fullerene derivatives (as the electron acceptor, A) with interpenetrating networks have attracted a myriad of attention for both academic and industrial sectors due to their premium features of flexibility, fabrication simplicity, low manufacturing costs, short energy payback time, and low environmental impact^1^,^2^,^3^. In the past few years, progresses have mainly focused on breaking the Shockley-Queisser limit by ameliorating device structures^4^,^5^,^6^ and developing novel low bandgap conjugated polymers^7^. Power conversion efficiencies (PCEs) over 10% from singe junction cells and as high as 12% from the tandem cells have been reported^8^,^9^. However, the fundamental question regarding energy losses during the photophysical process still remain obscure; particularly, the mechanisms of charge carrier recombination in BHJ PSCs are far from elucidated^10^.

As shown in Fig. 1, the charge carrier collection in BHJ PSCs includes the following steps/processes: formation of photo-induced excitons in D and A, respectively (1 & 1′); intra-molecular electron-hole recombination (2 & 2′); the excitons diffusion and dissociation at the D/A interface (3 & 3′)^1^,^2^,^3^; charge-transfer (CT) states generation and then dissociation into free charge carriers (electrons and holes) with an ultrafast quasi-adiabatic charge transfer process (4 & 4′); charge carriers that are transported through either D or A (5 & 5′) and then being collected by the respective electrodes (6 & 6′)^11^; the separated charge carriers may recombine with each other (7, geminate recombination) before dissociation; moreover, the separated charge carriers may also being collided and recombined (8, bimolecular recombination or non-geminate recombination) before collected by the respective electrodes (6 & 6′). The germinate and non-germinate recombinations are certainly responsible for the low PCEs in BHJ PSCs^2^,^12^.

On the other hand, the relative dielectric constant (*ε_r_*) of BHJ composite in PSCs is as low as 3, which is much smaller than that of typical inorganic counterparts (~10). The small dielectric constant results in strongly bounded Frenkel excitons with a diffusion length of ~10 nm for organic semiconductors rather than the Wannier excitons for inorganic semiconductors with a diffusion length of 10^4^ ~ 10^5^ nm^13^. Thus, in order to efficiently dissociate the photo-excited excitons in BHJ composite of PSCs, optimal phase separation with ~10 nm scale is required^1^. However, it is not easy to form a uniformly ideal ~10 nm interpenetrating phase separation in BHJ composite. As a result, most high efficiency PSCs were obtained by optimization of BHJ thin film morphology through huge processing effects. In addition, the traps and defects in BHJ composite also play a crucial role in exciton recombination^14^. Therefore, the challenge in forming uniformly ideal ~10 nm interpenetrating A–D phase separation and traps defects therein together with the low *ε_r_* of disordered organic materials induced various recombinations are responsible for approximately 50% efficiency loss among all loss pathways in BHJ PSCs^15^,^16^.

Studies from the transient photoconductivity, the time-delayed collection field, and the time-delayed dual pulse experiments have demonstrated that there is a competition process between the carrier sweep-out by the internal field and the loss of photogenerated carriers by recombination in BHJ PSCs^17^. Wherein the internal electric field with a value as high as 50 to 70 V/μm is required to ensure efficient charge collection at the short-circuit condition and in reverse bias in PSCs^18^,^19^. The asymmetrical electrode materials used in most of BHJ PSCs, however, afford a work-function difference of less than 2 eV producing an external electric field of ~20 V/μm (assuming the BHJ thickness is ~100 nm for typical device dimensions). This electric field is less efficient to sweep out photogenerated carriers and suppress charge carrier recombination in BHJ active layer^19^,^20^. Considering the insufficient electric field from the electrodes discussed above, a coercive electric field from magnetic nanoparticles (MNPs) show potential to strengthen the external electric field in BHJ PSCs.

In MNPs, a coercive electric field is produced among MNPs due to dipole interactions^21^. If the BHJ composite is incorporated with MNPs and then followed with an external magnetostatic field alignment, an orientated coercive electric field (*E*) will be created within BHJ composite (see in Fig. 2G). The *E* is described as: *E* = *(4πσf/ε)*^22^,^23^,^24^, where *ε* is the dielectric permittivity, *σ* is the surface charge density and *f* is the volume fraction of MNPs. For example, an additional *E* of 177.4 V/μm, which is at least 2 times larger than 50–70 V/μm, can be obtained by BHJ composite incorporated with 5% (by volume) of Fe_3_O_4_ MNPs. The details in calculation of *E* are described in Supplementary Information (SI 1). This additional coercive electric field is expected to enlarge the sweep-out rate of photogenerated carriers and suppress charge carrier recombination (both geminate and non-geminate); consequently resulting in enhanced PCEs in BHJ PSCs. In addition, these MNPs are also expected to influence the formation of thin film morphology of BHJ composite due to the motion of these MNPs under an external magnetostatic field^23^.

The *ε_r_* of Fe_3_O_4_ MNPs is 20, which is 5 times higher than that of BHJ composite (4) (*ε_r_* of poly(3-hexylthiophene) (P3HT) is 6.5 and *ε_r_* of phenyl-c61-butyric-acid-methyl ester (PC_61_BM) is 3.9, the *ε_r_* of P3HT:PC_61_BM BHJ composite is assumed to be ~4)^25^. The average *ε_r_* of BHJ composite incorporated with 5% (by volume) Fe_3_O_4_ MNPs can be enlarged by a factor of 20%^25^. Consequently, the Coulomb potential energy *E_c_*, 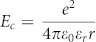 (where *e* is the charge of an electron, *ε_r_* is the relative dielectric constant of the surrounding medium, *ε*_0_ is the vacuum permittivity, and *r* is the electron-hole separation distance) of the CT state could be reduced due to enlarged *ε_r_* and optimized *r* (due to optimized BHJ film morphology). Moreover, the reduced *E_c_* will enlarge the total energy *U* of the CT state since the *U* is described as^26^:
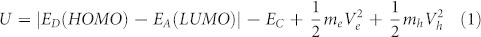
where *E_D_(HOMO)* and *E_A_(LUMO)* are the HOMO (highest occupied molecular orbital) energy level of D and the LUMO (lowest unoccupied molecular orbital) energy level of A; *V_e_* and *V_h_* are the electron and hole drifting velocities, respectively; *m_e_* and *m_h_* are the masses for electron and hole, respectively. In the eq. (1), the kinetic energies (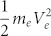 and 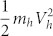) of charge carriers are increased due to the introduction of Fe_3_O_4_ MNPs dipole-induced coercive electric field, which is an additional electric field to drive the separated charge carriers to be transported through either D or A. As a result, decreased *E_C_* and increased kinetic energy would result in an enlarged *U* of the CT state. Therefore, it is unequivocal that the CT state becomes unstable which would facilitates the charge carrier dissociation^26^ resulting in an enlarged short-circuit current density (*J*_SC_) in PSCs^17^,^27^. Moreover, the direction of the dipolar moment produced by Fe_3_O_4_ MNPs is parallel in the presence of the vertically external magnetostatic field^22^,^23^,^24^. This parallel alignment could force Fe_3_O_4_ MNPs to be temporarily bound with the separated charge carriers in “ordered” structures, which facilitates the charge carrier to be transported to the respective electrodes (see Fig 2F & 2G and Fig. 4D). Therefore, the PSCs based on BHJ composite incorporated with Fe_3_O_4_ MNPs and then followed with a vertical external magnetostatic field alignment are expected to possess enhanced PCEs.

In order to verify above hypothesis, the PSCs fabricated by various BHJ composites, which are incorporated with Fe_3_O_4_ MNPs and then followed with an external magnetostatic field alignment, are investigated. The device architecture of PSCs is ITO/PEDOT:PSS/BHJ active layer/Calcium/Aluminum, where ITO is indium tin oxide, PEDOT:PSS is poly(ethylenedioxythiophene):poly(styrenesulfonate), BHJ active layer is BHJ composite incorporated with Fe_3_O_4_ MNPs. Here, we only report PSCs fabricated by PTB7-F20:PC_71_BM BHJ composite blended with 5% v/v Fe_3_O_4_ MNPs; the PSCs fabricated by other BHJ composites incorporated with Fe_3_O_4_ MNPs and the influence of Fe_3_O_4_ MNPs on the performance of PSCs are described in SI 2, SI 3 and SI 5. PFB7-F20 is fluorinated copolymer based on thieno[3,4-b]thiopehene coupled with 20% fluorine unit^28^ and PC_71_BM is phenyl-C_71_-butyric acid methyl ester. The molecular structures of PFB7-F20 and PC_71_BM are shown in Fig. 2A. The fabrication of PSCs incorporated with Fe_3_O_4_ MNPs is described in Figs. 2D to 2F and Fig. 2C (also SI 3). The device fabrication and characterization are described in experimental section. Fig. 2G illustrates that the direction of magnetic dipoles by Fe_3_O_4_ MNPs and the electric dipoles by an external electric field is in an antiparallel pattern. PSCs based on BHJ composite incorporated with Fe_3_O_4_ MNPs and then aligned by an external magnetostatic field alignment (represent as the PSCs-Fe_3_O_4_ W/H). PSCs based on BHJ composite incorporated with Fe_3_O_4_ MNPs without any external magnetostatic field alignment (represent as the PSCs-Fe_3_O_4_), and PSCs based on BHJ composite without Fe_3_O_4_ MNPs (represent as the control PSCs, Fig. 2B) were also fabricated and characterized for comparison.

The current densities versus voltage (*J–V*) characteristics of PSCs measured in the dark and under white light illumination are shown in Figs. 3A and 3B, respectively. All types of PSCs possess identical dark *J–V* characteristics with the rectification ratios larger than 10^4^, indicating that either Fe_3_O_4_ MNPs or an external magnetostatic field alignment did not alter the features of PSCs diodes^29^. Under white light illumination AM 1.5 with the light intensity of 100 mW/cm^2^ from solar simulator, the control PSCs exhibits a *J*_SC_ of 13.49 mA/cm^2^, an open-circuit voltage (*V*_OC_) of 0.65 V, a fill factor (*FF*) of 0.60, with a corresponding PCE of 5.26%; the PSCs-Fe_3_O_4_ yields a *J*_SC_ of 14.84 mA/cm^2^, a *V*_OC_ of 0.66 V, a FF of 0.69, a corresponding PCE of 6.76%; the PSCs-Fe_3_O_4_ W/H yields a *J*_SC_ of 16.20 mA/cm^2^, a *V*_OC_ of 0.67 V, a FF of 0.73, with a corresponding PCE of 7.93%. Both the PSCs-Fe_3_O_4_ and the PSCs-Fe_3_O_4_ W/H exhibit both enlarged *FF* and *J*_SC;_ more than 50% enhanced PCEs were observed from the PSCs-Fe_3_O_4_ W/H as compared with the control PSCs. Over 200 devices were fabricated and characterized; the deviation in PCEs is less than 10%.

The incident photon-to-electron conversion efficiency (IPCE) spectra for all PSCs were measured and the results are shown in Fig. 3C. The spectral responsibilities of all PSCs span from 350 to 850 nm. These observations are in good agreement with the absorption spectra observed from PTB7-F20:PC_71_BM BHJ composite thin films (SI and Fig. S3). Based on IPCE spectra, the estimated *J*_SC_ for the PSCs-Fe_3_O_4_ W/H, the PSCs-Fe_3_O_4_ and the control PSCs are 16.10 mA/cm^2^, 14.71 mA/cm^2^ and 13.39 mA/cm^2^, respectively. These estimated *J*_SC_ values are consistent with those observed from *J–V* characteristics (Fig. 3B). The PSCs-Fe_3_O_4_ W/H, the PSCs-Fe_3_O_4_ and the control PSCs show approximately 70%, over 60%, and approximately 60% IPCE values, respectively. The obviously enhanced IPCE values and PCEs demonstrate that both Fe_3_O_4_ MNPs and external magnetostatic field have certain degree influence on thin film morphology^1^,^2^,^3^, charge carrier mobilities^30^, dielectric constant of BHJ composite^25^, and charge carriers recombination in BHJ PSCs^11^.

In order to understand the underlying enhanced PCEs and IPCEs from the PSCs-Fe_3_O_4_ and the PSCs-Fe_3_O_4_ W/H, atomic force microscopy (AFM), transmission electron microscopy (TEM), and grazing-incidence small-angle scattering (GISAXS) are carried out to investigate the differences in thin film morphological diversities caused by either Fe_3_O_4_ MNPs or the effect of Fe_3_O_4_ MNPs with an external magnetostatic field alignment. The details in AFM, TEM, and GISAXS measurement and related results are described in SI 6 and Figs. S8–S10. Various fibrillar featured domains with a relatively high surface roughness of ~4.57 nm (SI 6, Fig. S8) were obtained from BHJ composite mixed with Fe_3_O_4_ MNPs and then followed by an external magnetostatic field alignment (represent as BHJ-Fe_3_O_4_ W/H); however, surface roughness of ~1.26 nm and ~1.15 nm were observed from BHJ composite mixed with Fe_3_O_4_ MNPs (represent as BHJ-Fe_3_O_4_) and BHJ composite, respectively. The surface of TEM images of BHJ-Fe_3_O_4_ W/H, BHJ-Fe_3_O_4_ and BHJ composite are almost identical (SI 6, Fig. S9). However, there are significant differences in cross-section TEM images. Figs. 4A and 4B show the cross-section TEM images of BHJ-Fe_3_O_4_ W/H and BHJ-Fe_3_O_4_. Evidently, Fe_3_O_4_ MNPs are randomly distributed in BHJ-Fe_3_O_4_, while Fe_3_O_4_ MNPs are aligned in certain orders in BHJ-Fe_3_O_4_ W/H. These aligned Fe_3_O_4_ MNPs are solely due to the magnetic dipole interaction^31^,^32^,^33^. Due to the magnetically and electrically anisotropic properties of Fe_3_O_4_ MNPs, *e.g.* Fe_3_O_4_ Janus particles^34^, the coercive electric field within Fe_3_O_4_ MNPs can be constrained in vertically direction by means of magnetically induced rotation and alignment of Fe_3_O_4_ MNPs, which originate from the magnetic dipole direction within an external magnetostatic field^31^. This coercive electric field makes Fe_3_O_4_ MNPs to be temporarily bound with the separated charge carriers in the ordered directions, which facilitates separated charge carriers to be transported to the respective electrodes. As a result, high PCEs and IPCEs are observed from the PSCs-Fe_3_O_4_ and the PSCs-Fe_3_O_4_ W/H.

GISAXS is further carried out to characterize the structural features with *d*-spacing on the domain size level (long range) in BHJ thin layer. Figs. 5A & 5B present the GISAXS patterns at the incident angle of 0.2° for BHJ-Fe_3_O_4_ and BHJ-Fe_3_O_4_ W/H and Fig. 5C presents the fitting curve of GISAXS pattern of BHJ-Fe_3_O_4_ W/H. The GISAXS patterns of BHJ composite and BHJ-Fe_3_O_4_ are almost identical and do not have any distinctive in-plane order, which indicate a random distribution of Fe_3_O_4_ MNPs inside BHJ active layer. However, in BHJ-Fe_3_O_4_ W/H, a diffraction peak along Q_y_ direction is located at 0.08 Å^−1^, which indicates an ordered, self-assembled Fe_3_O_4_ MNPs was formed (see Fig. 5B)^35^. Moreover, the value of 0.08 Å^−1^ corresponds to an interparticle spacing of 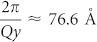 76.6 Å, where Q_y_ is a component of scattering vector. It is apparent that Fe_3_O_4_ MNPs were orientated in a certain orders within the BHJ interpenetrating network due to an external magnetostatic field alignment. Moreover, by controlling the kinetic alignment of Fe_3_O_4_ MNPs within BHJ composite, the thermodynamics of the D-A interpenetrating network should be affected^17^, consequence, as shown in SI Fig. S8, the BHJ-Fe_3_O_4_ W/H active layer shows premium morphology with more exquisite D-A separation of a uniform scale of ~10 nm, ensuring sufficient exciton dissociation. This refined phase-separation dimension indicates a larger interfacial area for efficient charge generation. In short, the magnetically induced film morphology rearrangement leads to an ordered and nanoscale optimized interpenetrating network, which facilitates charge carriers to be transported to the respective electrodes, simultaneously reduces the possibility of charge carrier recombination^4^,^10^. As a result, enhanced PCEs are observed from PSCs-Fe_3_O_4_ W/H.

The photo-electronic characteristics of PSCs are further investigated to confirm the effect of coercive electric field on charge carrier collection efficiency. Fig. 6A shows the photocurrent (*J*_ph_) versus the effect voltage (*V*_eff_) (*J*_ph_-*V*_eff_) characteristics of PSCs under AM 1.5 G illumination. At a large reverse voltage (*V*_eff_ = 1.9 V), *J*_ph_ is saturated for three different PSCs, suggesting that the photogenerated excitons are dissociated into free charge carriers and these charge carriers are collected by the electrodes without any residual non-geminate recombination^36^,^37^,^38^. As a result, the saturation current densities (*J*_sat_) are only dependent upon the amount of absorbed incident photon flux^37^. The maximum obtainable exciton generation rates are essentially the same for all three types of PSCs because the Fe_3_O_4_ MNPs contributed negligible absorption to BHJ composite (SI 4, Fig. S3). At *V*_eff_ = *V*_OC_ (*V*_OC_ = 0.65 V), the *J*_ph_/*J*_sat_ are 92.2%, 91.6% and 88.8% (*J*_sat_ is the reverse saturation photocurrent at *V*_eff_ = −1.9 V) for the PSCs-Fe_3_O_4_ W/H, the PSCs-Fe_3_O_4_, and the control PSCs, respectively. Interestingly, in the low effective voltage range, i.e. *V*_eff_ < 0.5 V, *J*_ph_-*V*_eff_ characteristics of these three types PSCs show distinct differences. At the maximum power output condition at *V*_eff_ = 0.2 V, *J*_ph_/*J*_sat_ are 84.6%, 83.1% for the PSCs-Fe_3_O_4_ W/H and the PSCs-Fe_3_O_4_, while it is only 78.7% for the control PSCs. Since the ratio of *J*_ph_/*J*_sat_ is the essential of exciton dissociation efficiency and charge carrier collection efficiency, a decreased *J*_ph_/*J*_sat_ suggests either reduced exciton dissociation efficiency or decreased charge carrier collection efficiency. The decreased charge carrier collection efficiency suggests that non-geminate recombination is dominated (compete over exciton-dissociation), resulting in a low *FF*. The charge carrier recombination in PSCs is manifested by the deviation of the photocurrent from the square-root dependence on effective voltage, which is one of the signatures of charge carrier recombination-limited photocurrent in PSCs^38^. The superior *J*_ph_-*V*_eff_ characteristics from the PSCs-Fe_3_O_4_ W/H clearly demonstrate the effect of Fe_3_O_4_ MNPs and external magnetostatic field alignment on reducing the geminate recombination at the low effective voltage, at which maximum power output condition of PSCs usually takes place. Such reduced geminate recombination in PSCs is probably originated from high charge carrier mobility of BHJ composite therein. Therefore, the enhancement in charge carrier diffusion and charge carrier transport are responsible for the distinctly different *J*_ph_/*J*_sat_ among all PSCs.

Light intensity-dependent efficiencies (*J*_SC_ and *V*_OC_) were further studied to confirm the effect of the coercive electric field on suppression of geminate and non-geminate recombinations in PSCs. In solar cells, if the mean drift length of the electron or hole (or both) is smaller than the thickness of photoactive layer, geminate recombination becomes considerable. Figs. 6B & 6C represent the steady-state light-intensity dependence of *J*_SC_ and *V*_OC_ for all PSCs. The PSCs-Fe_3_O_4_ W/H exhibits a near-linear dependence of *J*_SC_ with the light intensity, and a coefficient of *α* = 0.99 corresponding to the power law *J*_SC_ ∝ *I^α^*, where *I* is the light intensity. Both the PSCs-Fe_3_O_4_ and the control PSCs show slightly non-linear characteristics of *J*_SC_ versus *I* with a coefficient of *α* = 0.95 and *α* = 0.92, respectively. The different *α* values indicate that non-geminate recombinations are different in these three typed PSCs. The nearly linear dependence of *J*_SC_ is consistent with sweep-out at short circuit; however, this also indicates that non-geminate recombination is relatively weak^17^.

When PSCs are measured under illumination at open circuit voltage, the applied voltage equals to the difference between the quasi-Fermi-levels within the polymer and fullerene phase separated domains. The relations between *V*_OC_ and light intensity can be described as *V*_OC_ ∝ *S*ln(*I*), where *S* is the slope and *I* is the light intensity^38^. The fits for the PSCs-Fe_3_O_4_ W/H, the PSCs-Fe_3_O_4_ and the control PSCs are shown in Fig. 6C. The slope of *S* = 0.028, which is close to the value of KT/q (0.026), is observed from the PSCs-Fe_3_O_4_ W/H. This observation is consistent with the predictions of a drift-diffusion model with constant quasi-Fermi levels throughout the PSCs, indicating the geminate recombination is significantly suppressed in the PSCs-Fe_3_O_4_ W/H^17^. The slope of *S* = 0.032 is observed from the PSCs-Fe_3_O_4_, suggesting an alleviated geminate recombination compared with the control PSCs whose slope is 0.034^38^.

In PSCs, due to the low charge carrier mobility of disordered organic materials, charge carrier recombination becomes the dominant loss mechanism as the thickness of BHJ active layer increases. Fig. 6D presents PCEs versus the thickness of BHJ active layer. It was found that as the thickness of BHJ thin films increases from 120 nm to 260 nm, the PCEs from the control PSCs are significantly decreased from 5.2% to 4.5%; however, the PCEs from the PSCs-Fe_3_O_4_ decreased from 5.8% to 5.4%; while the PCEs from the PSCs-Fe_3_O_4_ W/H maintained almost the same value, around 7.0%. These results demonstrate that Fe_3_O_4_ MNPs and an external magnetostatic field alignment indeed can suppress the charge carrier recombination in the PSCs based on BHJ composite incorporated with Fe_3_O_4_ MNPs and then followed by an external magnetostatic field alignment.

In BHJ PSCs, the built-in electric field can be canceled at the condition of applied bias voltage (*V*_appl_) equals to *V*_OC_; at this condition, the photogenerated charge carriers in the active layer flowing toward the electrodes can be prevented^17^. As a result, the possibility of charge recombination at the D/A interface is increased to the maximum value. The impedance spectroscopy (IS) is carried out to monitor the detailed electrical properties of BHJ composite and/or the interface between each layer that cannot be observed by direct current measurement. The details of IS measurement is described in SI 7. In all PSCs, the difference in the resistance of PSCs solely comes from the CT resistance with BHJ composite active layer. Fig. 7 shows the Nyquist plot of PSCs at *V*_appl_ = *V*_OC_ and under 100 mW/cm^2^ from AM 1.5 G illumination. The plot of PSCs contains a semicircle which indicates that BHJ active layer is relatively homogeneous along the transport pathways without having discernible multiple interfacial boundaries^39^. At *V*_appl_ = *V*_OC_, the CT resistance of the control PSCs is ~83 Ω and this value decreases to ~58 Ω and ~32 Ω for the PSCs-Fe_3_O_4_ and the PSCs-Fe_3_O_4_ W/H, respectively. A significantly decreased CT resistance demonstrates that thin film morphologies are rearranged through PTB7-F20 crystallization and/or PC_71_BM aggregation^11^, which enhances the charge carrier transport and decreases the possibility of charge carrier recombination at the D/A interface in BHJ active layer. These observations are consistent with the film morphologies presented in AFM images (SI 6, Fig. S8) and are in good agreement with our hypothesis that an external magnetostatic field alignment can force Fe_3_O_4_ MNPs to create temporary “channels” for transporting separated charge carriers to the respective electrodes^25^.

Based on space charge limited current (SCLC) method, charge carrier mobilities of PTB7-F20 and PC_71_BM are investigated to verify the accuracy of IS and to understand high FF from the PSCs-Fe_3_O_4_ W/H and the PSCs-Fe_3_O_4_ as well. Single charge carrier devices were fabricated and Mott-Gurney law was applied to estimate either electron mobility of PCBM or hole mobility of PTB7-F20. The details of single charge carrier devices fabrication and the method using Mott-Gurney law to estimate charge carrier mobilities are described in SI 8. As shown in Fig. 8, hole mobilities (μ_h_) of 4.43 × 10^−4^ cm^2^/Vs, 2.38 × 10^−4^ cm^2^/Vs and 1.09 × 10^−4^ cm^2^/Vs are observed from the PTB7-F20 incorporated with Fe_3_O_4_ MNPs and then followed with an external magnetostatic field alignment, the PTB7-F20 incorporated with Fe_3_O_4_ MNPs and pristine PTB7-F20, respectively. Electron mobilities (μ_e_) of 5.25 × 10^−4^ cm^2^/Vs, 2.33 × 10^−4^ cm^2^/Vs and 1.18 × 10^−4^ cm^2^/Vs are observed from the PC_71_BM incorporated with Fe_3_O_4_ MNPs and then followed with an external magnetostatic field alignment, the PC_71_BM incorporated with Fe_3_O_4_ MNPs and pristine PC_71_BM, respectively. Both enlarged hole mobility of PTB7-F20 and electron mobility of PC_71_BM are observed from PTB7-F20 and PC_71_BM incorporated with Fe_3_O_4_ MNPs and then followed with an external magnetostatic field alignment, respectively. Consequently, reduced charge carrier recombination and enlarged *J*_SC_ and FF are observed from the PSCs-Fe_3_O_4_ W/H. While the microscopic origin of enhanced mobility remains uncertain at this point, we speculate that aligned dipoles by an external magnetostatic field may facilitate charge carriers to escape shallow traps; thus, improving their mobilities^37^,^38^.

In conclusion, we have investigated the influence of magnetic nanoparticles and an external magnetostatic field on the PCEs of PSCs. The optimization of BHJ thin film morphology, suppression of charge carrier′s recombination and enhancement in free carrier collection result in more than 50% enhanced efficiency from the PSCs fabricated by BHJ composite blended with Fe_3_O_4_ magnetic nanoparticles and then followed with an external magnetostatic field alignment. Our work represents an evolution of PSCs that applications of magnetic nanoparticles and magnetostatic field alignment to BHJ composite have proven to be an extraordinarily effective way to enhance power conversion efficiency of PSCs.

## Methods

### Materials

PTB7-F20, PC_61_BM and PC_71_BM were provided by 1-Material Inc. PBDTTT-C-T was provided by Prof. Y. F. Li and Prof. J. H. Hou in the Institute of Chemistry at the Chinese Academy Science, P. R. China. P3HT was purchased from Rekie Metal Inc. All materials used as received without further purification. Fe_3_O_4_ MNPs toluene solution was purchased from Sigma-Aldrich. The size of Fe_3_O_4_ MNPs is ~5 nm.

### Device Preparation

**Figure 2:**
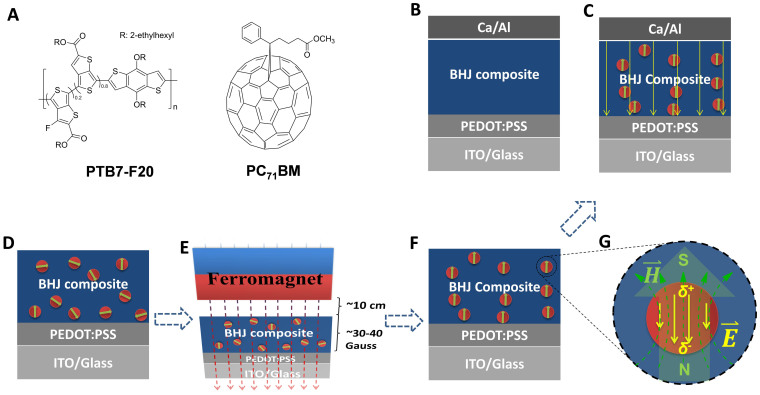
(A) The molecular structures of PTB7-F20 and PC_71_BM; (B) the conventional device structure of PSCs without incorporating any Fe_3_O_4_ magnetic nanoparticels (MNPs); (C) the conventional device structure of PSCs incorporated with Fe_3_O_4_ MNPs and aligned by an external magnetostatic field; (D)–(F) the fabrication procedures of PSCs incorporated with Fe_3_O_4_ MNPs and aligned by an external magnetostatic field; (D) BHJ active layer incorporated with Fe_3_O_4_ MNPs was spin-coated on PEDOT:PSS coated ITO substrate; (E) a ferromagnet was suspend above the surface of BHJ composite incorporated with Fe_3_O_4_ MNPs layer. The magnetic intensity is ~30–40 G and the distance between the ferromagnet and BHJ composite layer is ~10 cm; (F) oriented Fe_3_O_4_ MNPs inside BHJ active layer by an external magnetostatic field. In pre-devices; (G) Drawing of partial enlargement of Fe_3_O_4_ MNP in (C), showing an antiparallel relation between the magnetic dipole (caused by the Fe_3_O_4_ crystal inside the particle) and electric dipole (caused by the difference of charge density between the inside Fe_3_O_4_ and outside organic coater).

The PSCs architecture is ITO/PEDOT:PSS/BHJ active layer/Calcium/Aluminum, where ITO is indium-doped tin oxide, PEDOT:PSS is poly(ethylenedioxythiophene):poly(styrenesulfonate), and the BHJ active layer is polymer:fullerene blend including PTB7-F20:PC_71_BM, PBDTTT-C-T:PC_71_BM and P3HT:PC_61_BM. ITO coated glass slides are firstly cleaned with detergent, followed by ultrasonic washing in deionized water, acetone, isopropanol, and subsequently dried in an oven overnight. The ITO is treated with oxygen plasma for 40 min to modify the work function of ITO before spin-casting a ~30 nm thick PEDOT:PSS on top of it. The PEDOT:PSS coated ITO glasses are then backed on hotplate at 150°C for 10 min in the air. After that, PEDOT:PSS coated ITO glasses are transferred into the glove box of N_2_ atmospheres. Then three different types of active layer are solution-processed on top of PEDOT:PSS layer with same thickness of ~200 nm. For the control PSCs, the active layer was spin-coated from a binary solution of polymer and fullerene in *o*-DCB with a concentration of 10 mg/mL. For PSCs-Fe_3_O_4_ and PSCs-Fe_3_O_4_ W/H, the active layers were spin-coated from a ternary solution of polymer, fullerene and small amount of Fe_3_O_4_ MNPs. (e.g. PTB7-F20:PC_71_BM BHJ composite (1:1.5, w/w, 10 mg/mL in *o*-DCB) mixed with Fe_3_O_4_ MNPs (1 mg/mL in toluene) by a volume ratio of 5%) (Fig. 2D). During the processing for PSCs-Fe_3_O_4_ W/H, an external magnetic field is applied to align the MNPs inside the active layer. The direction of magnetostatic field is perpendicular to the ITO substrate. The magnetostatic field is generated by square magnet (C750, 3/4'' Cube, Licensed NdFeB, the intensity of the magnetostatic field is 30 ~ 40 Gauss, the distance to the ITO substrates is ~10 cm) (Fig. 2E). Its direction and intensity is manipulated by tuning the magnet pole direction (North and South) as well as adjusting the distance between these two square magnets, respectively. By using such specific magnet, the distance and intensity on the surface of active layer is controlled to ~10 cm and ~400 G, respectively. Finally, top electrode (Ca and Al) are sequentially deposited onto the active layer under a pressure of ca. 5 × 10^−6^ mbar (Fig. 2C).

### Characterization and Measurement

The *J–V* curves characteristics are measured using a Keithley 2400 Source Measure Unit. The solar cells are characterized using a Newport Air Mass 1.5 Global (AM 1.5 G) full spectrum solar simulator with irradiation intensity of 100 mW/cm^−2^. The light intensity is measured by a monosilicon detector (with KG-5 visible color filter) which is calibrated by National Renewable Energy Laboratory (NREL). Device masks were made using laser beam cutting technology and had well-defined areas of 0.16 or 0.045 cm^2^.

GISAXS experiments were done at the Advanced Photon Source at Argonne National Laboratory. And the IS is obtained using a HP 4194A Impedance/gain-phase analyzer. All the devices are measured under 100 mW/cm^2^ AM 1.5 G illumination, with an oscillating voltage of 10 mV and frequency of 1 Hz to 1 MHz. All PSCs are held at their respective open circuit potentials obtained from the *J–V* measurements, while the IS spectra are recorded.

## Author Contributions

K.W., C.Y., C.L., X.W.H. and C.H.H. conducted the experiments. S.C. involved deep discussion of the project and IS measurement. X.G. thought of the idea and supervised the project.

## Supplementary Material

Supplementary Information Supporting Information

## Figures and Tables

**Figure 1:**
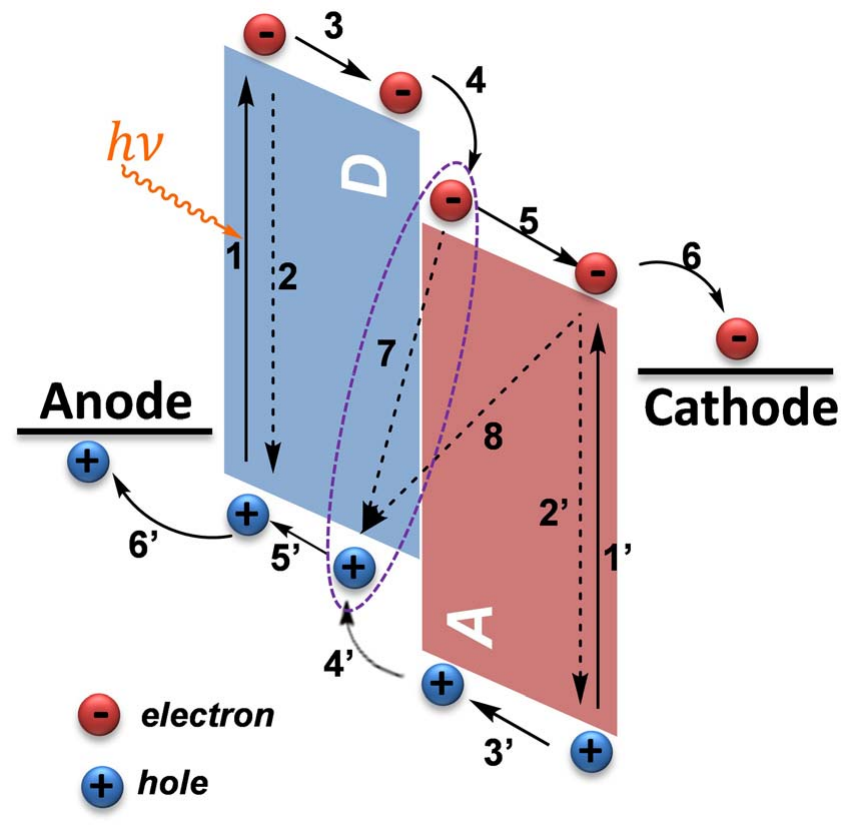
The operational principle of bulk-heterojunction polymer solar cells: formation of photo-induced excitons in D and A, respectively (1 & 1′); intra-molecular electron-hole recombination (2 & 2′); the excitons diffusion and dissociation at the D/A interface (3 & 3′); generation of charge-transfer (CT) states and these CT states dissociate into free charge carriers (electrons and holes) with an ultrafast quasi-adiabatic charge transfer process (4 & 4′); charge carriers that are transported through either D or A (5 & 5′) and then collected by the respective electrodes (6 & 6′); the separated charge carriers may recombine with each other (7, geminate recombination) before dissociation; the separated charge carriers may also collide and be recombined (8, bimolecular recombination or non-geminate recombination) before being collected by the respective electrodes (6 & 6′).

**Figure 3:**
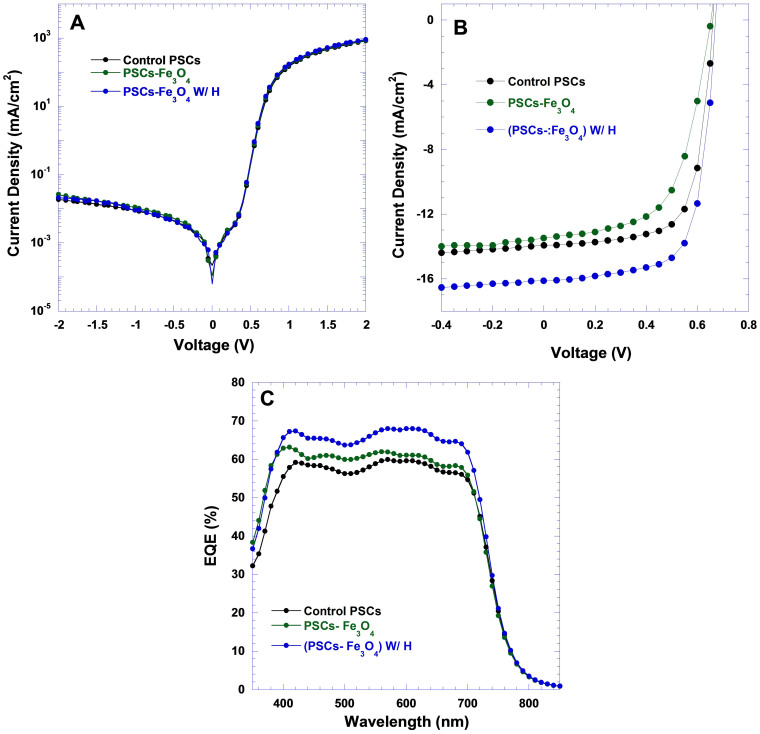
(A) *J*–*V* characteristics of PSCs in the dark, (B) *J*–*V* characteristics of PSCs measured under 100 mW/cm^2^ AM 1.5 G illumination, and (C) IPCE spectra of BHJ PSCs versus wavelength.

**Figure 4:**
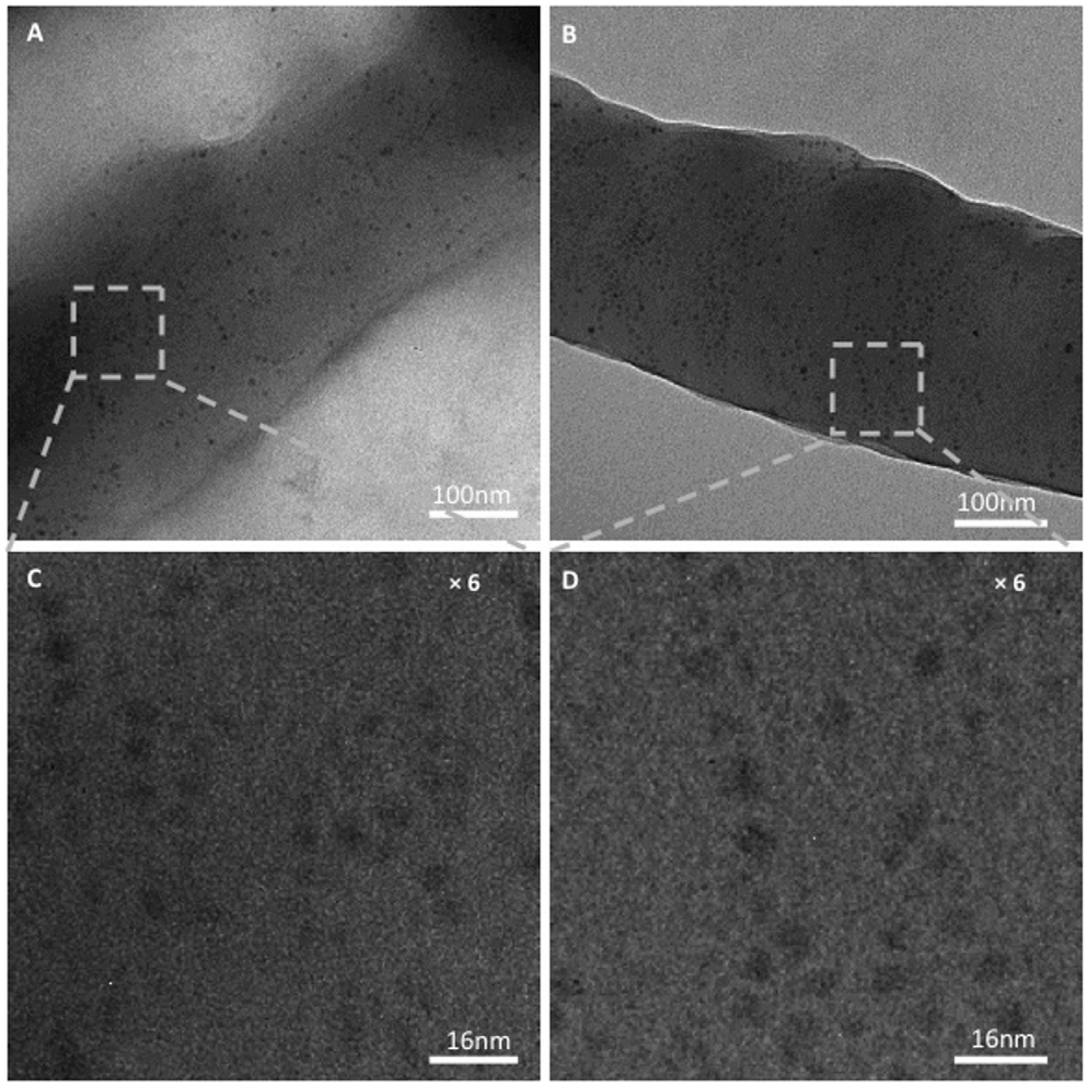
Cross-section TEM images of BHJ composite incorporated with Fe_3_O_4_ MNPs (A) without an external magnetostatic field alignment and (B) with an external magnetostatic field alignment, (C) and (D) are 6 times enlarged views of (A) and (B), respectively.

**Figure 5:**
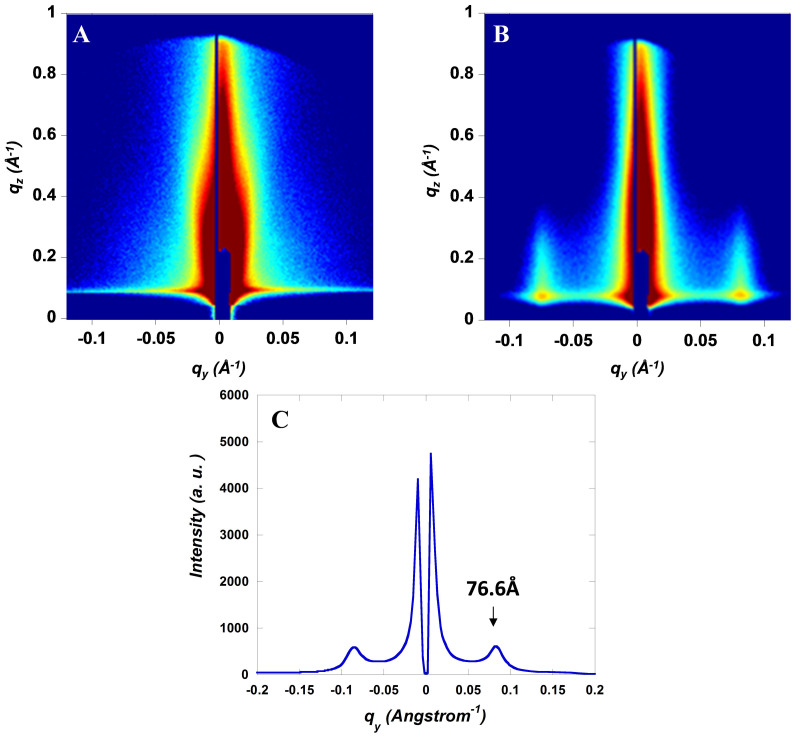
Grazing-incidence small-angle scattering (GISAXS) pattern at the incident angle of 0.2° for (A) BHJ composite incorporated with Fe_3_O_4_ magnetic nanoparticles (MNPs) without any external magnetostatic field treatment and (B) BHJ composite incorporated with Fe_3_O_4_ MNPs and followed with an external magnetostatic field alignment. (C) Fitting curve of GISAXS pattern of BHJ composite incorporated with Fe_3_O_4_ MNPs and followed with an external magnetostatic field alignment.

**Figure 6:**
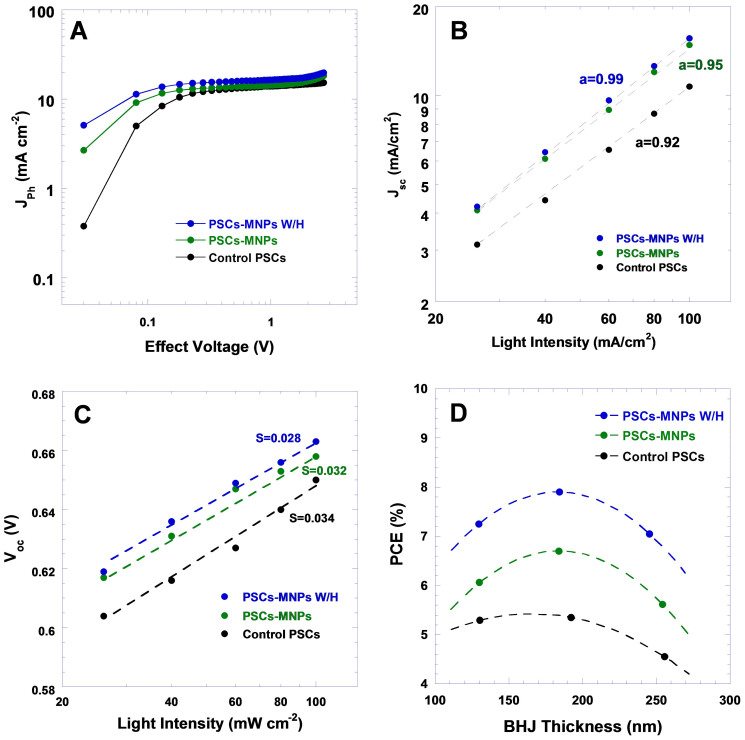
(A) Photocurrent density versus effective voltage (*J*_ph_ – *V*_eff_) characteristics of PSCs under constant incident light intensity (AM 1.5 G, 100 mW/cm^2^), (B) photocurrent density (*J*_sc_) versus the light intensity, and (C) open-circuit voltage (*V*_oc_) versus of the light intensity. (The lines in (B) represent fits to the expression *J*_sc_ ∝ *I*^α^ while the lines in (C) represent fits to the expression *V*_oc_ = SIn(I/I_0_), and (D) Device efficiencies (%) versus of BHJ film thickness (nm).

**Figure 7:**
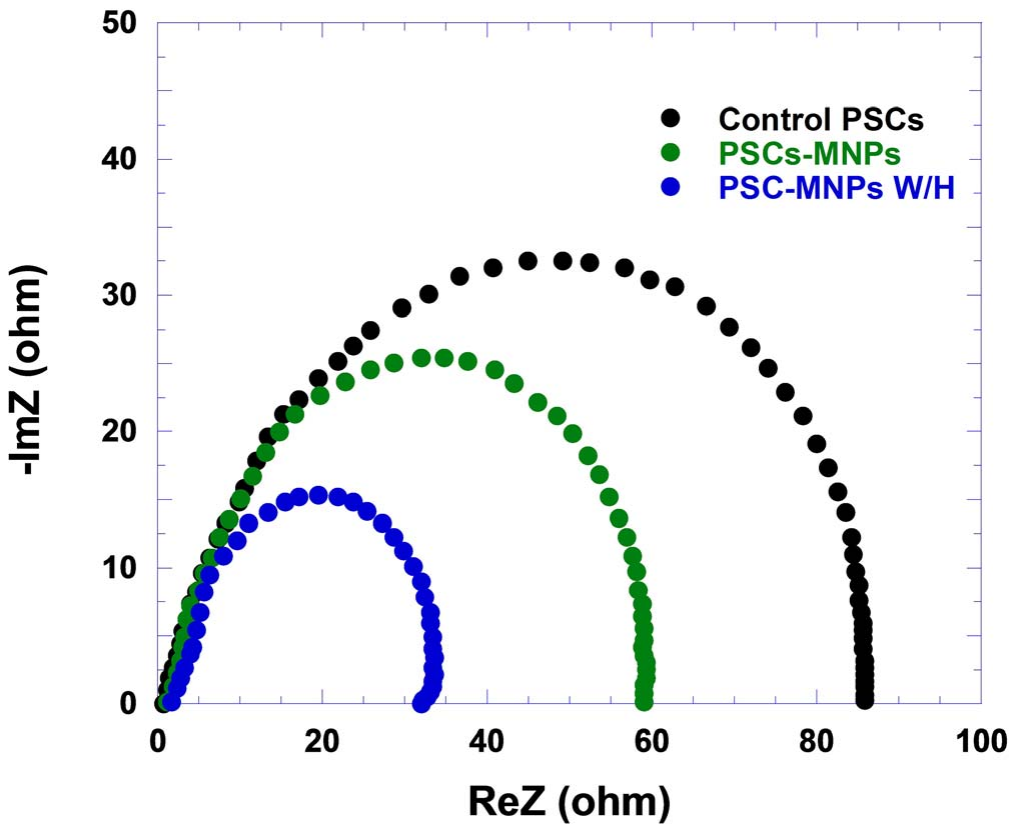
Nyquist plots at *V* = *V*_oc_ for PSCs under light irradiation.

**Figure 8:**
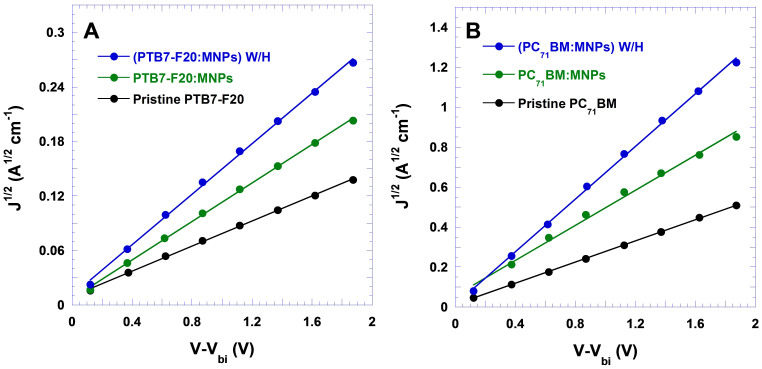
*J*^1/2^ versus *V*–*V*_bi_ for (A) hole-only diode made by PTB7-F20 and (B) electron-only diodes made by PC_71_BM.
