# Supplementary material for: Effects of Magnetic Nanoparticles and External Magnetostatic Field on the Bulk Heterojunction Polymer Solar Cells
Source: Sci Rep. 2015 Mar 18;5:9265. doi: 10.1038/srep09265 (PMC4363869; doi:10.1038/srep09265)
Supplement: Supplementary Information — Supporting Information [file srep09265-s1.docx]

#### Supplementary Information

Effects of Magnetic Nanoparticles and External Magnetostatic Field on the Bulk Heterojunction Polymer Solar Cells

Kai Wang,^1^ Chao Yi,^1^ Chang Liu,^1^ Xiaowen Hu,^1,2^ Steven Chuang,^1^ and Xiong Gong*^1,2^

1. College of Polymer Science and Polymer Engineering, The University of Akron, Akron, OH 44325, USA
2. State Key Laboratory of Luminescent Materials and Devices, South China University of Technology, Guangzhou, 510640, P. R. China

Table of Contents

Effects of Magnetic Nanoparticles and External Magnetostatic Field on the Bulk Heterojunction Polymer Solar Cells 1

SI 1. Coercive Electric Field Intensity 2

SI 2. Materials Used for Fabrication of BHJ PSCs 3

SI 2.1. Electron donor polymers 3

SI 2.2. Electron accepter fullerene derivatives 3

SI 2.3. Fe_3_O_4_ MNPs 3

SI 3. BHJ PSCs Fabrications 3

SI 3.1. The control PSCs 3

SI 3.2. The PSCs-Fe_3_O_4_ 4

SI 3.3. The PSCs-Fe_3_O_4_ W/H 5

SI 4. Absorption Spectra 6

SI 4.1. Film preparation 6

SI 4.2. Instrument 6

SI 4.3. Absorption spectra 6

SI 5. PSCs Characterization 6

SI 5.1. PTB7-F20:PC_71_BM system 7

SI 5.2. PBDTTT-C-T:PC_71_BM system 7

SI 5.3. P3HT:PC_61_BM system 8

SI 5.4 The influence of Fe_3_O_4_ MNPs concentrations on the efficiency of PSCs 9

SI 6. Thin Film Morphology 10

SI 6.1. AFM measurement 10

SI 6.2. TEM measurement 11

SI 6.3. GISAXS measurement 12

SI 7. Impedance Spectra (IS) 12

SI 7.1. Device preparation 12

SI 7.2. Instrument 13

SI 7.3. Results 13

SI 8. Charge Carrier Mobility 13

SI 8.1. Hole mobility 14

SI 8.2. Electron mobility 14

References 16

# SI 1. Coercive Electric Field Intensity

Fig. S1 describes the statuses of Fe_3_O_4_ magnetic nanoparticles (MNPs) in the bulk heterojunction (BHJ) active layer before and after an external magnetostatic field alignment. It is evident that Fe_3_O_4_ MNPs (possessing magnetic dipoles) are randomly distributed in the BHJ composite without external magnetostatic field alignment.^[1]^ After aligned by an external magnetostatic field, Fe_3_O_4_ MNPs can be tuned in a certain order.^[1]^ Moreover, the direction of the magnetic dipole moment within each MNP is antiparallel in the presence of the externally applied magnetostatic field.^[2]^

The electric dipole induced coercive electric field within MNPs is described as follows:^[3]^

$E=-\frac{Z_{0}}{4\pi}k^{2}(n\times m)\frac{e^{ikr}}{r}(1-\frac{1}{ikr})$ (1)

by using the classical dipole-field model.^[4-10]^ The dipoles of a concentration *f* in medium could create an average field:

$E=\frac{4\pi}{\varepsilon}\sigmaƒ$ (2)

where ε is the dielectric permittivity (the relative permittivity (*ε_r_*) of Fe_3_O_4_ MNPs is ~20);^[7]^ σ is the pyroinduced surface charge density (5.0 μC/cm^2^ for Fe_3_O_4_ MNPs);^[8]^ and *f* is the volume fraction occupied by the dipoles in the medium.

The coercive field is estimated to be 3.55 × 10^3^ × *f* V/μm. If a small volume fraction, for example, if *f* = 0.05, the coercive field will be177.4 V/μm.

# SI 2. Materials Used for Fabrication of BHJ PSCs

## SI 2.1. Electron donor polymers

The electron donor polymers under our investigation are: PTB7-F20, PBDTTT-C-T (thieno[3,4-b]thiophene (TT) and benzo[1,2-b:4,5-b´]dithiophene (BDT) alternating units)^[10]^ and P3HT. The molecular structures of these polymers are shown in Fig. S2. PTB7-F20 ^[14]^ was provided by 1-Material Inc. PBDTTT-C-T was provided by Prof. Y. F. Li and Prof. J. H. Hou in the Institute of Chemistry at the Chinese Academy Science, P. R. China. P3HT was purchased from Rekie Metal Inc. All materials used as received without further purification.

## SI 2.2. Electron accepter fullerene derivatives

#### The electron acceptors under our investigation are: [6,6]-phenyl-C71-butyric acid methyl ester (PC_71_BM) and [6,6]-phenyl-C61-butyric acid methyl ester (PC_61_BM). Both PC_61_BM and PC_71_BM are purchased from 1-Material Inc. and used as received without further purification. The molecular structures of PC_61_BM and PC_71_BM are shown in Fig. S2.

## SI 2.3. Fe_3_O_4_ MNPs

Fe_3_O_4_ MNPs toluene solution was purchased from Sigma-Aldrich. The size of Fe_3_O_4_ MNPs is ~5 nm.

# SI 3. BHJ PSCs Fabrications

## SI 3.1. The control PSCs

The PSCs architecture is ITO/PEDOT:PSS/BHJ active layer/Calcium/Aluminum, where ITO is indium-doped tin oxide, PEDOT:PSS is poly(ethylenedioxythiophene): poly(styrenesulfonate), and the BHJ active layer is polymer:fullerene blend including PTB7-F20:PC_71_BM, PBDTTT-C-T:PC_71_BM and P3HT:PC_61_BM.

ITO coated glass slides are firstly cleaned with detergent, followed by ultrasonic washing in deionized water, acetone, isopropanol, and subsequently dried in an oven overnight. The ITO is treated with oxygen plasma for 40 min to modify the work function of ITO before spin-casting a ~30 nm thick PEDOT:PSS on top of it. The PEDOT:PSS coated ITO glasses are then backed on hotplate at 150 ℃ for 10 min in the air. After that, PEDOT:PSS coated ITO glasses are transferred into the glove box of N_2_ atmospheres. Then a solution of polymer:fullerene is spin-coated on top of PEDOT:PSS layer. The concentration of each polymer system is 10 mg/mL in *o*-DCB. After that, the sample is immediately transferred into a petri dish and kept till the film is dried in the glove box. The thickness of the active layer is around ~200 nm, e.g. for system of PTB7-F20:PC_71_BM the thickness of active blend is approximately 180 nm. Finally, top electrodes (Ca and Al) are sequentially deposited onto the active layer in a pressure of ca. 5× 10^-5^ mbar. The active area of PSCs is measured to be 0.045 cm^2^.

## SI 3.2. The PSCs-Fe_3_O_4_

The PSCs architecture is ITO/PEDOT:PSS/BHJ active layer/Calcium/Aluminum, the BHJ active layer is polymer:fullerene composite incorporated with Fe_3_O_4_ MNPs including PTB7-F20:PC_71_BM blended with Fe_3_O_4_ MNPs, PBDTTT-C-T:PC_71_BM blended with Fe_3_O_4_ MNPs and P3HT:PC_61_BM blended with Fe_3_O_4_ MNPs.

ITO coated glass slides are firstly cleaned with detergent, followed by ultrasonic washing in deionized water, acetone, isopropanol, and subsequently dried in an oven overnight. The ITO is treated with oxygen plasma for 40 min to modify the work function of ITO before spin-casting a ~30 nm thick PEDOT:PSS on top of it. The PEDOT:PSS coated ITO glasses are then backed on hotplate at 150 ℃ for 10 min in the air. After that, PEDOT:PSS coated ITO glasses are transferred into the glove box of N_2_ atmospheres. A solution of polymer:fullerene composite (e.g. PTB7-F20:PC_71_BM BHJ composite (1:1.5, w/w, 10 mg/mL in *o*-DCB)) mixed with Fe_3_O_4_ MNPs (1 mg/mL in toluene) by a volume ratio of 5% is spin-coated on top of PEDOT:PSS layer. After that, the sample is immediately transferred into a petri dish and kept till dried in the glove box. The thickness of the active layer is around ~200 nm, e.g. for system of PTB7-F20:PC_71_BM doped with MNPs, the thickness of active blend is approximately 180 nm. Finally, top electrode (Ca and Al) are sequentially deposited onto the active layer under a pressure of ca. 5 × 10^-5^ mbar. The active area of PSCs is measured to be 0.045 cm^2^.

## SI 3.3. The PSCs-Fe_3_O_4_ W/H

The PSCs architecture is ITO/PEDOT:PSS/BHJ active layer/Calcium/Aluminum, the BHJ active layer is polymer:fullerene composite incorporated with Fe_3_O_4_ MNPs including PTB7-F20:PC_71_BM blended with Fe_3_O_4_ MNPs, PBDTTT-C-T:PC_71_BM blended with Fe_3_O_4_ MNPs and P3HT:PC_61_BM blended with Fe_3_O_4_ MNPs. During the processing, an external magnetic field is applied to align the MNPs inside the active layer. The external magnetostatic field is offered by a magnet. And by changing the distance from the surface of active layer and the surface gauss level of the magnet, the magnetic field intensity can be tuned from 1 Gauss to 600 Gauss. The direction of the external magnetostatic field can be tuned by changing the orientation of the magnet.

ITO coated glass slides are firstly cleaned with detergent, followed by ultrasonic washing in deionized water, acetone, isopropanol, and subsequently dried in an oven overnight. The ITO is treated with oxygen plasma for 40 min to modify the work function of ITO before spin-casting a ~30 nm thick PEDOT:PSS on top of it. The PEDOT:PSS coated ITO glasses are then backed on hotplate at 150 ℃ for 10 min in the air. After that, PEDOT:PSS coated ITO glasses are transferred into the glove box of N_2_ atmospheres. A solution of polymer:fullerene composite (e.g. PTB7-F20:PC_71_BM BHJ composite (1:1.5, w/w, 10 mg/mL in *o*-DCB)) mixed with Fe_3_O_4_ MNPs (1 mg/mL in toluene) by a volume ratio of 5% is spin-coated on top of PEDOT:PSS layer. After that, the sample is immediately transferred into a petri dish. An external magnetostatic field is applied to the wet film. The direction of magnetostatic field is perpendicular to the ITO substrate. The magnetostatic field is generated by square magnet (C750, 3/4'' Cube, Licensed NdFeB). Its direction and intensity is manipulated by tuning the magnet pole direction (North and South) as well as adjusting the distance between these two square magnets, respectively. By using such specific magnet, the distance and intensity on the surface of active layer is controlled to ~10 cm and ~400 G, respectively. The thickness of the active layer is around ~200 nm, the same as the control devices described above. Finally, top electrode (Ca and Al) are sequentially deposited onto the active layer under a pressure of ca. 5 × 10^-5^ mbar. The active area of PSCs is measured to be 0.045 cm^2^ as well.

# SI 4. Absorption Spectra

## SI 4.1. Film preparation

All the solid films are spin-coated on quartz substrates. The quartz substrates are cleaned with detergent, followed by ultrasonic washing in deionized water, acetone, isopropanol, and subsequently dried in an oven overnight. The thin films are made as those for fabrication of PSCs.

## SI 4.2. Instrument

Absorption spectra were measured using a HP 8453 spectrophotometer.

## SI 4.3. Absorption spectra

The normalized absorption spectra of thin films are shown in Fig. S3. No obvious difference is observed in mostly absorption wavelength range from these three thin films, except that relative stronger absorption is found from the films of BHJ composite incorporated with Fe_3_O_4_ MNPs. This may be due to the high refractive index of Fe_3_O_4_ magnetic nanoparticles, which results in a high optical absorption in organic hybrid active layer.^[9]^

#

# SI 5. PSCs Characterization

For the following three different materials systems, the highest average PCE all comes from the PSC-Fe_3_O_4_ W/H. During the processing, we tried the external magnetostatic field with different magnetic field direction and intensity (from 1 Gauss to 600 Gauss with the corresponding distance from ~30 cm to ~0.5 cm). While the highest PCE comes from the device using a magnetostatic field with a vertical direction and an intensity of 30-40 Gauss with a corresponding distance of ~10 cm from magnet to active layer. By changing the direction of the magnetostatic field, the device performance of PSC-Fe_3_O_4_ W/H is equivalent to that of PSC-Fe_3_O_4_, while after using the magnetostatic field with a vertical direction but different intensity, the PSC-Fe_3_O_4_ W/H shows higher PCE than PSC-Fe_3_O_4_. For a stronger H, the NPs might be driven to the top or bottom of the active layer while a weaker H cannot offer a strong enough magnetic force to align the NPs inside the BHJ composite.

## SI 5.1. PTB7-F20:PC_71_BM system

The *J-V* curves characteristics are measured using a Keithley 2400 Source Measure Unit. The solar cells are characterized using a Newport Air Mass 1.5 Global (AM 1.5G) full spectrum solar simulator with irradiation intensity of 100 mW/cm^-2^. The light intensity is measured by a monosilicon detector (with KG-5 visible color filter) which is calibrated by National Renewable Energy Laboratory (NREL).

The *J-V* characteristics of the PSCs measured in dark are shown in Fig. S4A. The rectification ratios of the PSCs are larger than 10^4^ indicating that Fe_3_O_4_ magnetic nanoparticles did not alter the features of PSCs diodes. All the devices exhibit similar turn-on voltages, implying that the built-in potential (*V*_bi_) across the devices are similar. The *J-V* characteristics of the PSCs measured under simulated AM 1.5 illumination of 100 mW/cm^2^ are shown in Fig. S4B. The PSCs performance parameters are summarized in Table S1. The PSCs-Fe_3_O_4_ W/H exhibits *J*_sc_ of 16.20 mA/cm^2^, *V*_oc_ of 0.67 V, *FF* of 0.73, yielding PCE of 7.93 %. The PSCs-Fe_3_O_4_ exhibits *J*_sc_ of 14.84 mA/cm^2^, *V*_oc_ of 0.66 V, *FF* of 0.69, yielding PCE of 6.76 %. The control PSCs only exhibits *J*_sc_ of 13.49 mA/cm^2^, *V*_oc_ of 0.65 V, *FF* of 0.60, yielding PCE of 5.26 %. ~ 50 % enhanced PCE is observed from the PSCs-Fe_3_O_4_ W/H as compared with the control PSCs.

## SI 5.2. PBDTTT-C-T:PC_71_BM system

The current density (J)-voltage (V) curves characteristics are measured using a Keithley 2400 Source Measure Unit. The solar cells are characterized using a Newport Air Mass 1.5 Global (AM 1.5G) full spectrum solar simulator with irradiation intensity of 100 mW/cm^-2^. The light intensity is measured by a monosilicon detector (with KG-5 visible color filter) which is calibrated by National Renewable Energy Laboratory (NREL).

The *J-V* characteristics of the PSCs measured in dark are shown in Fig. S5A. The rectification ratios of the PSCs are larger than 10^4^ indicating that Fe_3_O_4_ magnetic nanoparticles did not alter the features of PSCs diodes. All the devices exhibit similar turn-on voltages, implying that the built-in potential (V_bi_) across the devices are similar. The *J-V* characteristics of the PSCs measured under simulated AM 1.5 illumination of 100 mW/cm^2^ are shown in Fig. S5B. The PSCs performance parameters are summarized in Table S2. The PSCs-Fe3O4 W/H exhibits *J*_sc_ of 14.87 mA/cm^2^, *V*_oc_ of 0.80 V, *FF* of 0.58, yielding PCE of 6.90 %. The PSCs-Fe_3_O_4_ exhibits *J*_sc_ of 13.13 mA/cm^2^, *V*_oc_ of 0.80 V, *FF* of 0.58, yielding PCE of 6.09 %. The control PSCs only exhibits *J*_sc_ of 10.59 mA/cm^2^, *V*_oc_ of 0.78 V, *FF* of 0.53, yielding PCE of 4.39 %. ~ 57 % enhanced PCE is observed from the PSC-Fe_3_O_4_ W/H as compared with the control PSCs.

## SI 5.3. P3HT:PC_61_BM system

The *J-V* curves characteristics are measured using a Keithley 2400 Source Measure Unit. The solar cells are characterized using a Newport Air Mass 1.5 Global (AM 1.5G) full spectrum solar simulator with irradiation intensity of 100 mW/cm^-2^. The light intensity is measured by a monosilicon detector (with KG-5 visible color filter) which is calibrated by National Renewable Energy Laboratory (NREL).

The current density-voltage (*J-V*) characteristics of the PSCs measured in dark were shown in Fig. S6A. The rectification ratios of the PSCs are more than 10^4^ indicating that Fe_3_O_4_ magnetic nanoparticles incorporated with BHJ composite did not alter the features of PSCs diodes. All the devices exhibited similar turn-on voltages, implying that the built-in potential (V_bi_) across the devices are similar. The *J-V* characteristics of the PSCs measured under simulated AM 1.5 illumination of 100 mW/cm^2^ were shown in Fig. S6B. The PSCs performance parameters were summarized in Table S1. The PSC made by P3HT:PC_61_BM blended with Fe_3_O_4_ MNPs followed with an external magnetostatic field alignment, exhibits *J*_sc_ of 10.95 mA/cm^2^, *V*_oc_ of 0.58 V, *FF* of 0.66, yielding PCE of 4.05%. The PSC made by P3HT:PC_61_BM blended with Fe_3_O_4_ nanoparticles exhibits *J*_sc_ of 9.19 mA/cm^2^, *V*_oc_ of 0.58 V, *FF* of 0.63, yielding PCE of 3.35 %. The PSC made by P3HT:PC_61_BM only exhibits *J*_sc_ of 7.32 mA/cm^2^, *V*_oc_ of 0.58 V, *FF* of 0.61, yielding PCE of 2.60 %. ~ 55% enhanced PCEs were observed from the PSCs made by P3HT:PC_61_BM blended with Fe_3_O_4_ magnetic nanoparticles followed with an external magnetostatic field alignment as compared with the PSCs made by P3HT:PC_61_BM.

## SI 5.4 The influence of Fe_3_O_4_ MNPs concentration on efficiency of PSCs

The relationship between the concentration of Fe_3_O_4_ MNPs and the device performance for the P3HT:PC_60_BM system is shown in Fig. S7. The optimal concentration of MNPs is 5% by volume. And by adding 1%-7% of MNPs into active layer, all the PSCs-Fe_3_O_4_ and PSCs-Fe_3_O_4_ W/H show higher *J_SC_* and PCE than the control device. However, after adding 9% MNPs, the device performance decrease largely and worse than that of control device. To point out, all the devices share the similar value of *V_OC_* and FF, while the *J_SC_* shows the regularity in Fig. S7, resulting in the differences in PCE. For the PSCs-Fe_3_O_4_, when the amount of MNPs increases from 1% to 5%, the device performance firstly enhance, while further increase the concentration of MNPs to 7%, the device performance decreases. When adding 9% MNPs, the PCE of the PSCs-Fe_3_O_4_ is even worse than the control device.

Similarly, for the PSCs-Fe_3_O_4_ W/H, the differences in PCE due to the MNPs concentration are larger compared with the PSCs-Fe_3_O_4_. And all the PSCs-Fe_3_O_4_ W/H show higher PCE and *J_SC_* than those of the PSCs-Fe_3_O_4_ except for the devices with 9% MNPs. Firstly, for smaller amount of MNPs, the MNPs induced additional electric field may not be strong enough compared with that of the 5% MNPs-PSCs-Fe_3_O_4_ W/H; and for the devices with higher concentration of MNPs, the introduction of the larger number of MNPs might influence the morphology of the active layer. According the Equation (2), the concentration is proportional to the coercive electric field strength. With a stronger coercive electric field, the possibility for exciton dissociation is larger. However, adding the MNPs will also cause the morphological change in the BHJ interpenetrating network, which might be either good or bad for the device performance. And the decrease of PCE in high MNPs concentration device might result from the decreased D-A interface area. Secondly, for the abnormal phenomena in PSCs with 9% MNPs, the PSCs-Fe_3_O_4_ W/H show worse performance compared with PSCs-Fe_3_O_4_. The reason behind might be the aggregation of MNPs inside the active layer since the amount of the particles is larger and the system entropy increases, making them easier to gather together.

# SI 6. Thin Film Morphology

## SI 6.1. AFM measurement

### Thin film preparation

All the BHJ composite solution without and with Fe_3_O_4_ MNPs are the same as those used for fabrication of PSCs. The PEDOT:PSS coated ITO-glass is used as substrate. The thin film preparations are the same as those for PSCs.

### Instrument

Tapping-mode atomic force microscopy (AFM) is carried out on a NanoScope NS3A system (Digital Instrument) to characterize the surface morphologies.

### Results

The nanoscale morphologies of the active layers are studied using tapping-mode AFM. Surface topography (left) and phase image (right) are shown in Fig. S8. Surface roughness values measured from the topography images are ~ 1.15 nm, ~ 1.26 nm, and ~ 4.57 nm for thin film of BHJ composite (Fig. S8A), thin film of BHJ composite incorporated with Fe_3_O_4_ (Fig. S8B) and thin film of BHJ composite incorporated with Fe_3_O_4_ magnetic nanoparticles followed with an external magnetostatic field alignment (Fig. S8C), respectively. As shown in Fig. S8, very different morphologies are observed for these three films in their phase images. For thin film of BHJ composite incorporated with Fe_3_O_4_ magnetic nanoparticles, the interpenetrating network and the fibrillar features are observed from the topography images, whereas domains with different shapes are observed. The different morphologies of these films suggest that the interactions between PTB7-F20 and PC_71_BM might be different. We speculate that the morphology different origins from the interaction between PTB7-F20 and PC_71_BM, which affected by the Fe_3_O_4_ magnetic nanoparticles.

## SI 6.2. TEM measurement

### Thin Film preparation

All the BHJ composite solution without and with Fe_3_O_4_ MNPs are the same as those used for fabrication of PSCs. The PEDOT:PSS coated ITO-glass is used as substrate. The thin film preparations are the same as those for PSCs.

### Instrument

Bright field TEM images are recorded on a JEOL-1230 microscope with an accelerating voltage of 120 kV. Ultra-thin film is prepared by microtoming using a Reichert Ultracut S (Leica) ultra-cryomicrotome machine.

### Results

The thin films on ITO glass are floated with water and collected with a needle, and dried in air for 24 hours to remove moisture. These films are cut into slides with a blade. One piece of the sample is embedded with embedding agent (Epo-Fix Embedding Resin: Epo-Fix Hardener, 25:3, w/w). The agent is cross linked under room temperature for 24 hours. Ultra thin specimen (typically 100 nm) is prepared by microtoming using a Reichert Ultracut S (Leica) ultra-cryomicrotome machine.

As shown in Fig. S9, the interpenetrating networks of thin film of BHJ composite (Fig. S9A) and thin film of BHJ composite incorporated with Fe_3_O_4_ magnetic nanoparticles (Fig. S9B) are not well developed, and the Donor (D)-Acceptor (A) domains are difficult to distinguish. For the thin film of BHJ composite incorporated with Fe_3_O_4_ magnetic nanoparticles followed with an external magnetostatic field alignment (Fig. S9C), the morphology of the interpenetrating D-A networks become clearer and easily visible. The changes in morphology will result in a large interfacial area for efficient charge generation.

## SI 6.3. GISAXS measurement

### Thin film preparation

All the BHJ composite solution without and with Fe_3_O_4_ MNPs are the same as those used for fabrication of PSCs. The PEDOT:PSS coated ITO-glass is used as substrate. The thin film preparations are the same as those for PSCs.

### Instrument

GISAXS experiments were done at the Advanced Photon Source at Argonne National Laboratory.

### Results

The GISAXS patterns of BHJ composite and the BHJ composite incorporated with Fe_3_O_4_ MNPs are almost identical and do not have any distinctive side maximums, which indicate a random distribution of Fe_3_O_4_ MNPs inside the BHJ active layer. However, in BHJ composite incorporated with Fe_3_O_4_ MNPs and then followed an external magnetostatic field alignment, the side peaks is located at 0.08 Å^-1^, which indicates an ordered self-assembled Fe_3_O_4_ MNPs was formed.

# SI 7. Impedance Spectra (IS)

## Device preparation

The device architectures for IS measurement is ITO/PEDOT:PSS/BHJ active layer/Ca/Al, where BHJ active layer is PTB7-F20:PC_71_BM or PTB7-F20:PC_71_BM incorporated with Fe_3_O_4_. The procedures of devices fabrication are the same as those for PSCs. The thicknesses of BHJ active layers are 180 nm.

## Instrument

The IS is obtained using a HP 4194A Impedance/gain-phase analyzer. All the devices are measured under 100 mW/cm^2^ AM 1.5 G illumination, with an oscillating voltage of 10 mV and frequency of 1 Hz to 1 MHz. All PSCs are held at their respective open circuit potentials obtained from the *J-V* measurements, while the IS spectra are recorded.^11^

## Results

At *V*_appl_ = *V*_oc_, the CT resistance of the control PSCs is ~ 83 Ω, and this value decreases to ~ 58 Ω and ~ 32 Ω for the PSCs-Fe_3_O_4_ and the PSCs-Fe_3_O_4_ W/H, respectively. A significantly decreased CT resistance demonstrates that morphology at the nanoscale is rearranged through PTB7-F20 crystallization and/or PC_71_BM aggregation, which enhances the charge carrier transport and decrease the possibility of charge carrier recombination at the D/A interface in BHJ active layer.

# SI 8. Charge Carrier Mobility

Space charge limited current (SCLC) is utilized to investigate the charge carriers mobilities. We made single charge carrier devices and applied Mott-Gurney law to estimate electron and hole mobilities, respectively

The mobilities (hole or electron) are determined through fitting *J-V* curves to the equation

$$J=\frac{8}{9}\varepsilon_{0}\varepsilon_{r}\mu\frac{V^{2}}{L^{3}},$$

where *ε*_r_ is the relative permittivity of the material; *ε*_0_ is the permittivity of free space, and the *ε*_r_ is 3 for typical conjugated polymers; L is the thickness of the active layer, and *µ* is the zero-field mobility.

## SI 8.1. Hole mobility

### Device preparation

The hole mobility is measured by using hole-only device with a device architecture of ITO/PEDOT:PSS/active layer/MoO_3_/Al.^12^ The device preparation procedures are the same as those for PSCs, but the active layer is only PTB7-F20 without and with Fe_3_O_4_ MNPs. ~15 nm MoO_3_ is evaporated as electron block layer in hole-only device. The thicknesses of active layers are 180 nm and the active device area is measured to be 0.045 cm^2^.

### Instrument

Hole mobility is measured by taking current-voltage current in the range of 0-2 V under 100 mW/cm^2^ AM 1.5 G illumination. The current density (*J*)-voltage (*V*) curves characteristics are measured using a Keithley 2400 Source Measure Unit.

### Results

Hole mobilities (*µ*_h_) of 4.43 × 10^-4^ cm^2^/Vs, 2.38 × 10^-4^ cm^2^/Vs and 1.09 × 10^-4^ cm^2^/Vs are observed from the PTB7-F20 incorporated with Fe_3_O_4_ MNPs and then followed with an external magnetostatic field alignment, the PTB7-F20 incorporated with Fe_3_O_4_ MNPs and pristine PTB7-F20, respectively.

## SI 8.2. Electron mobility

### Device preparation

The device architecture for electron-only mobility measurement is ITO/Al/active layer /Al.^13^ The device preparation procedures are same as those for PSCs, but the active layer is only PC_71_BM without and with Fe_3_O_4_ magnetic nanoparticles. The thicknesses of active layers are 90 nm and the active device area is measured to be 0.045 cm^2^.

### Instrument

Hole mobility is measured by taking current-voltage current in the range of 0-2 V under 100 mW/cm^2^ AM 1.5 G illumination. The current density (*J*)-voltage (*V*) curves characteristics are measured using a Keithley 2400 Source Measure Unit.

### Results

Electron mobilities (*µ*_e_) of 5.25 × 10^-4^ cm^2^/Vs, 2.33 × 10^-4^ cm^2^/Vs and 1.18 × 10^-4^ cm^2^/Vs are observed from the PC_71_BM incorporated with Fe_3_O_4_ MNPs and then followed with an external magnetostatic field alignment, the PC_71_BM incorporated with Fe_3_O_4_ MNPs and pristine PC_71_BM, respectively.

# References

1. Zhang, W.-X., Sun, J.-F., Bai, T.-T., Wang, C.-Y., Zhuang, K.-H., Zhang, Y. & Gu, N. Quasi-one-dimensional assembly of magnetic nanoparticles induced by a 50 Hz alternating magnetic field. *ChemPhysChem* **11,** 1867-1870 (2010).

2. Mørup, S., Hansen, M. F. & Frandsen, C. Magnetic interactions between nanoparticles. *Beilstein J. Nanotechnol.* **1,** 182-190 (2010).

3. Jackson J. D. in *Classical Electrodynamics*, (Wiley, (New York), ed. 3, 1999)

4. Hwang, J. G., Zahn, M., O’Sullivan, F. M., Pettersson, L. A. A., Hjortstam, O. & Liu, R.-S. Effects of nanoparticle charging on streamer development in transformer oil-based nanofluids. *J. Appl. Phys.* **107,** 014310-014326 (2010).

5. Nalwa, K. S., Carr, J. A., Mahadevapuram, P. C., Kodali, H. K., Bose, S., Chen, Y.-Q., Petrich, J. W., Ganapathysubramanian, B. & Chaudhary, S. Enhanced charge separation in organic photovoltaic films doped with ferroelectric dipoles. *Energy Environ. Sci.* **5,** 7042-7049 (2012).

6. Shvydka, D. & Karpov, V. Nanodipole photovoltaics. *Appl. Phys. Lett.* **92,** 053507-053509 (2008).

7. Lide, D. R. in *CRC Handbook of Chemistry and Physics*, (CRC press, 2012)

8. Ziese, M., Esquinazi, P. D., Pantel, D., Alexe, M., Nemes, N. M. & Garcia-Hernández, M. Magnetite (Fe_3_O_4_): a new variant of relaxor multiferroic? *J. Phys.: Condens. Matter* **24,** 086007-086014 (2012).

9. Huo, L.-J., Zhang, S.-Q., Guo, X., Xu, F., Li, Y. F. & Hou, J. H. Replacing alkoxy groups with alkylthienyl groups: a feasible approach to improve the properties of photovoltaic polymers. *Angew. Chem. Int. Ed.* **50,** 9697-9702 (2011).

10. Zhang, H., Han, J.-S. & Yang, B. Structural fabrication and functional modulation of nanoparticle–polymer composites. *Adv. Funct. Mater.* **20,** 1533-1550 (2010).

11. Kim, J. S., Chung, W. S., Kim, K., Kim, D. Y., Paeng, K.-J., Jo, S. M. & Jang, S.-Y. Performance optimization of polymer solar cells using electrostatically sprayed photoactive layers. *Adv. Funct. Mater.* **20,** 3538-3546 (2010).

12. Li, B.-X., Chen, J. S., Yang, D.-Z. & Ma, D.-G. A comparative investigation of trap-limited hole transport properties in organic bulk heterojunctions. *Semicond. Sci. Tech.* **26,** 115006-115010 (2011).

13. Chen, M.-R., Fu, W.-F., Shi, M.-M., Hu, X.-L., Pan, J.-Y., Ling, J., Li, H.-Y. & Chen, H.-Z. An ester-functionalized diketopyrrolopyrrole molecule with appropriate energy levels for application in solution-processed organic solar cells. *J. Mater. Chem. A* **1,** 105-111 (2013).

14. Wang H.-X., *et al*. Fine-tuning of fluorinated thieno[3,4-b]thiophene copolymer for efficient polymer solar cells. *J. Phys. Chem. C* **117**, 4358-4363 (2013).


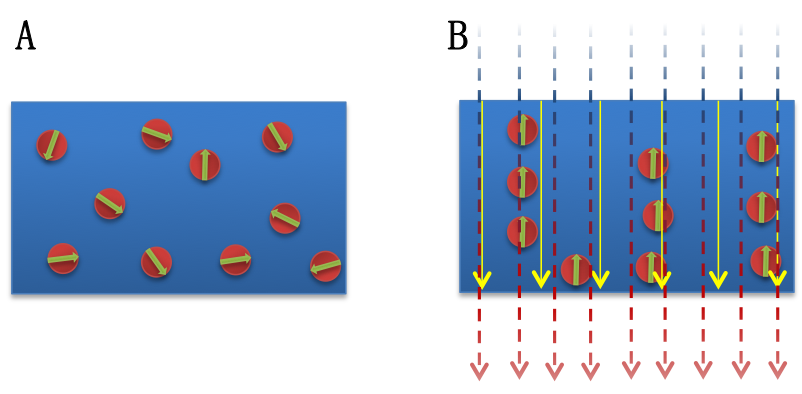


**Figure S1**. The statuses of Fe_3_O_4_ MNPs within the BHJ active layer before (A) and after (B) external magnetostatic field alignment.

**Figure S2.** Chemical structures of (A) electron donor materials: PTB7-F20, PBDTTT-C-T, P3HT and (B) electron acceptor materials: PC71BM, PC61BM.

**Figure S3.** Absorption spectra of PTB7-F20:PC_71_BM BHJ composite film (denoted as BHJ composite), BHJ composite incorporated with Fe_3_O_4_ magnetic nanoparticles (denoted as BHJ composite:Fe_3_O_4_), BHJ composite incorporated with Fe_3_O_4_ magnetic nanoparticles followed with an external magnetostatic field alignment (denoted as (BHJ Composite:Fe_3_O_4_) W/ H).

**Figure S4.** *J-V* characteristics of PSCs measured in dark (A) and under 100 mW/cm^2^ AM 1.5 G illumination (B).

**Table S1.** PSCs performance parameters for the PTB7-F20:PC_71_BM BHJ system.

| **PSCs** | ***V*_OC_ (V)** | ***J*_SC_ (mA/cm^2^)** | ***FF*** | **PCE (%)** |
| --- | --- | --- | --- | --- |
| Control PSCs | 0.65 | 13.49 | 0.60 | 5.26 |
| PSCs-Fe_3_O_4_ | 0.66 | 14.84 | 0.69 | 6.76 |
| PSCs-Fe_3_O_4_ W/H | 0.67 | 16.20 | 0.73 | 7.93 |

**Table S2.** PSCs performance parameters for the PBDTTT-C-T:PC_71_BM BHJ system.

| **PSCs** | ***V*_OC_ (V)** | ***J*_SC_ (mA/cm^2^)** | ***FF*** | **PCE (%)** |
| --- | --- | --- | --- | --- |
| Control PSCs | 0.78 | 10.59 | 0.53 | 4.39 |
| PSCs-Fe_3_O_4_ | 0.80 | 13.13 | 0.58 | 6.09 |
| PSCs-Fe_3_O_4_ W/H | 0.80 | 14.87 | 0.58 | 6.90 |

**Figure S5**. *J-V* characteristics of PSCs measured in dark (A) and under 100 mW/cm^2^ AM 1.5 G illumination (B).

**Table S2.** PSCs performance parameters for the P3HT:PC_61_BM BHJ system.

| **PSCs** | ***V*_OC_ (V)** | ***J*_SC_ (mA/cm^2^)** | ***FF*** | **PCE (%)** |
| --- | --- | --- | --- | --- |
| Control PSCs | 0.58 | 7.32 | 0.61 | 2.60 |
| PSCs-Fe_3_O_4_ | 0.58 | 9.19 | 0.63 | 3.35 |
| PSCs-Fe_3_O_4_ W/H | 0.58 | 10.95 | 0.66 | 4.05 |

**Figure S6.** (A) *J-V* characteristics of the PSCs measured in dark (B) under 100 mW/cm^2^ AM 1.5 G illumination.

**Figure S7.** The relationship between the PSCs device performance and the concentration of Fe_3_O_4_ MNPs for the P3HT:PC_60_BM system.


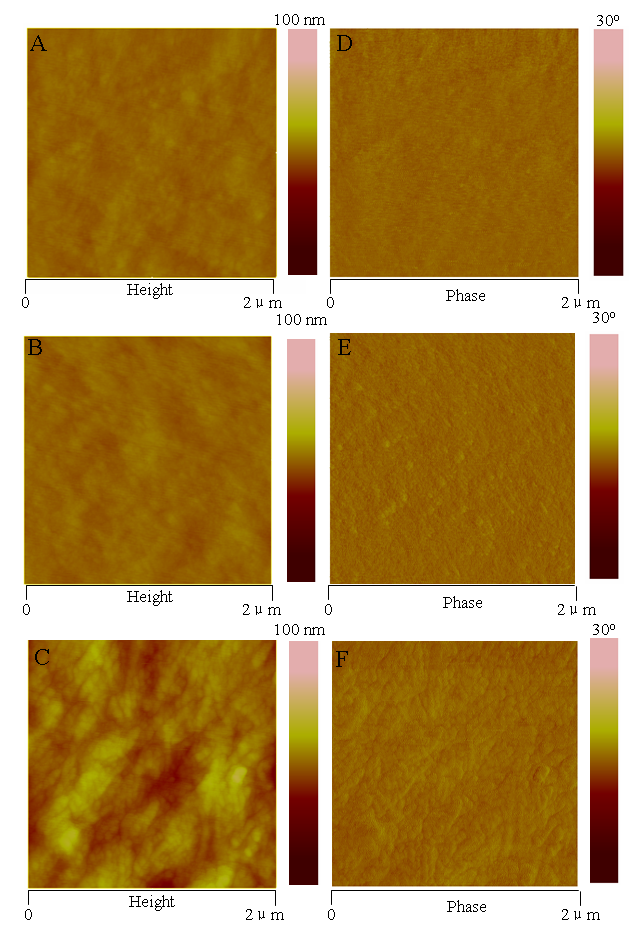


**Figure S8.** Tapping-mode AFM topography and phase images of films (A and D) PTB7-F20:PC_71_BM BHJ composite, (B and E) BHJ composite incorporated with Fe_3_O_4_ MNPs, and (C and F) BHJ composite incorporated with MNPs followed with an external magnetostatic field alignment.

**
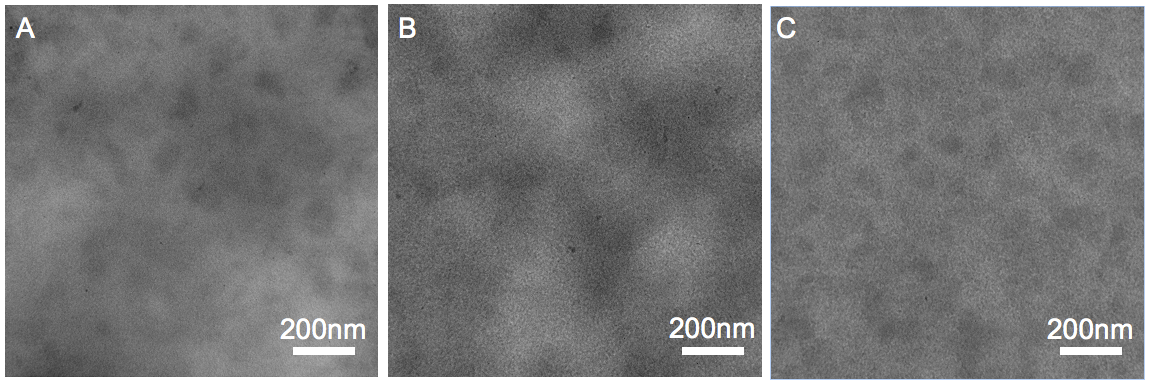
**

**Figure S9.** TEM bright-field images of films (A) PTB7-F20:PC_71_BM BHJ composite, (B) BHJ composite incorporated with Fe_3_O_4_ MNPs, and (C) BHJ composite incorporated with Fe_3_O_4_ MNPs followed with an external magnetostatic field alignment.


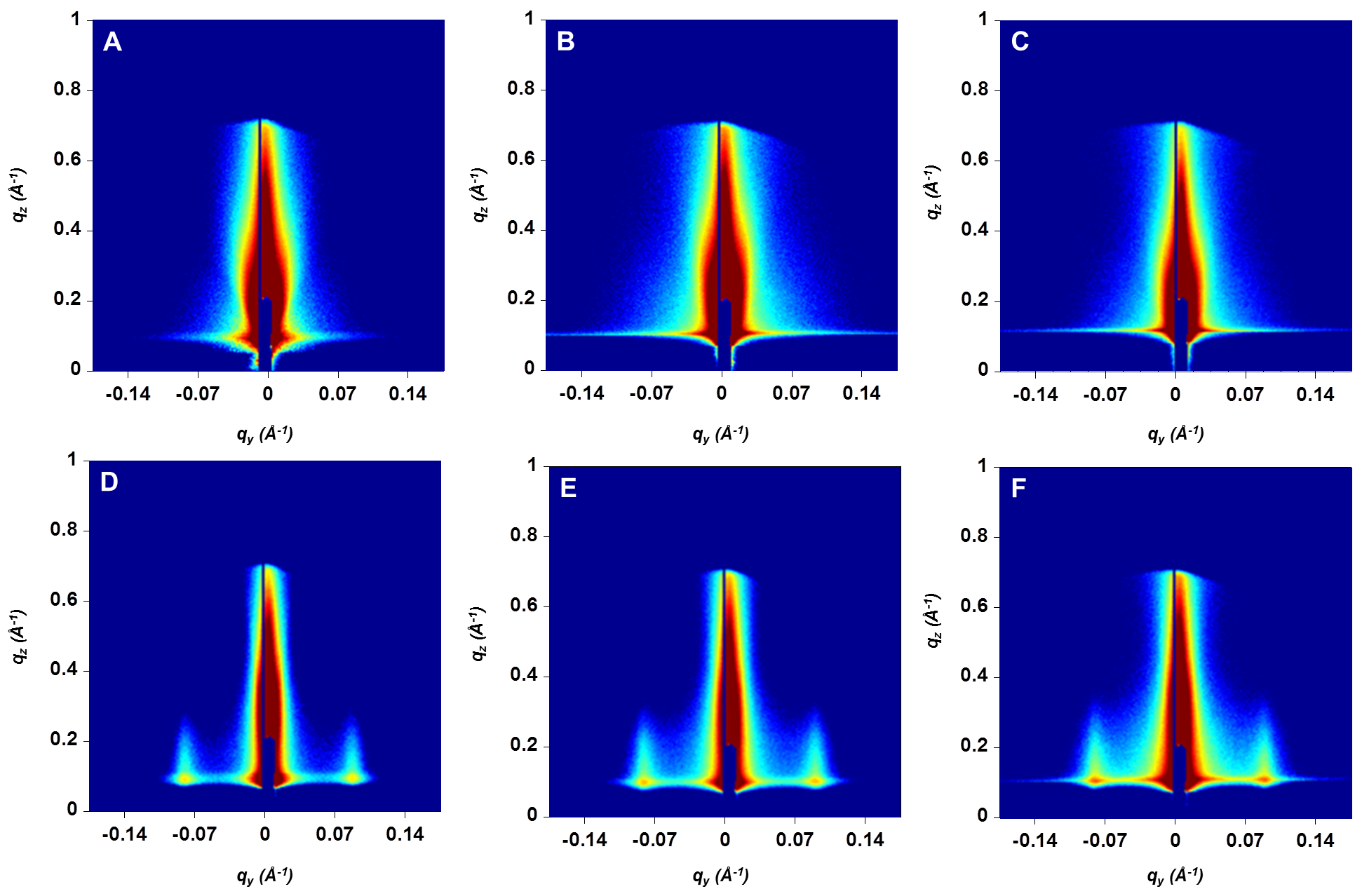


**Figure S10.** Grazing-incidence small-angle scattering (GISAXS) pattern of control BHJ composite without MNPs with an incident angle of (A) 0.15^o^ (B) 0.2 ^o^ (C) 0.23 ^o^ and experimental BHJ composite incorporating MNPs with an external magnetostatic field alignment with an incident angle of (D) 0.15^o^ (E) 0.2 ^o^ (F) 0.23 ^o^.

**
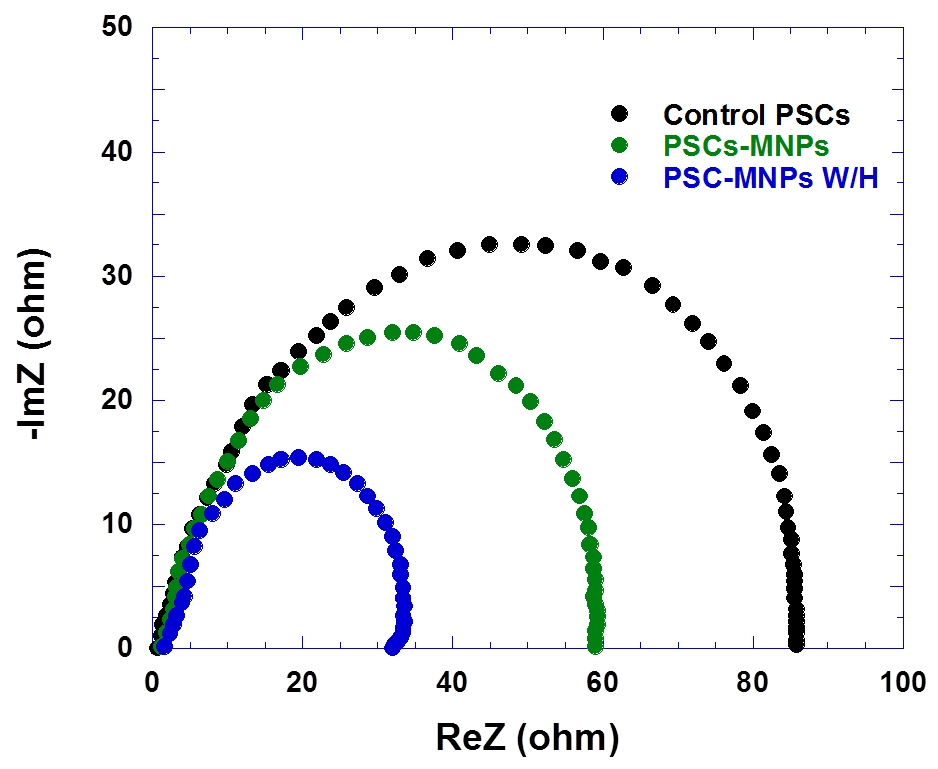
**

**Figure S11.** Nyquist plots at *V* = *V*_oc_ for PSCs under light irradiation. Note: the control PSCs (black), the PSCs-Fe_3_O_4_ (green), and the PSCs-Fe_3_O_4_ W/H (blue).


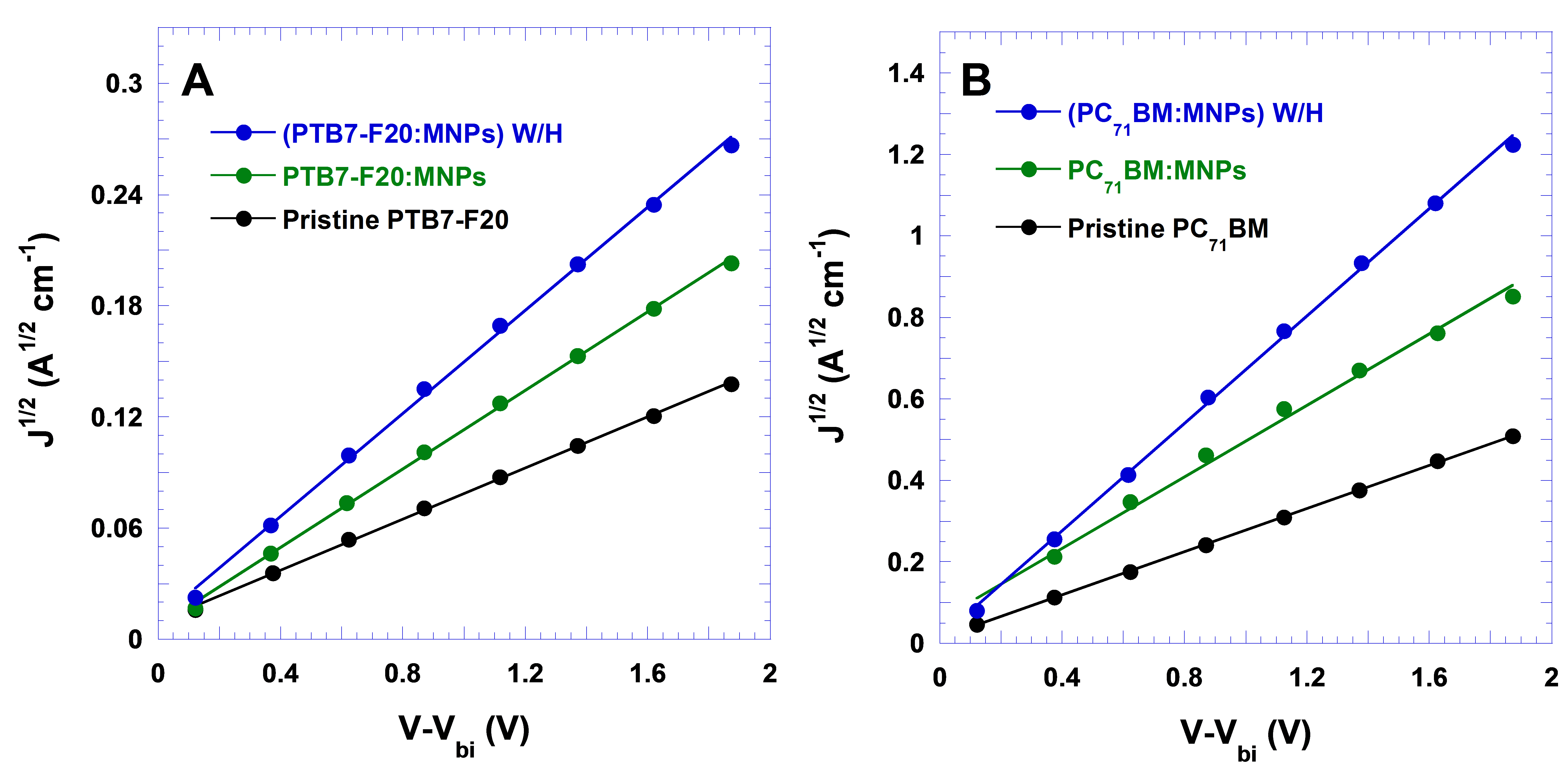


**Figure S12.** *J*^1/2^ versus *V*-*V*_bi_ for **(A)** hole-only diode made by PTB7-F20 and **(B)** electron-only diodes made by PC_71_BM. Note, the diodes without Fe_3_O_4_ magnetic nanoparticles (black), the diodes with Fe_3_O_4_ magnetic nanoparticles but without an external magnetostatic field alignment (green), and the diodes with both Fe_3_O_4_ magnetic nanoparticles and an external magnetostatic field alignment (blue).
